# Association of HLA-A, B, DRB1 alleles and haplotypes with HIV-1 infection in Chongqing, China

**DOI:** 10.1186/1471-2334-9-201

**Published:** 2009-12-12

**Authors:** Xia Huang, Hua Ling, Wei Mao, Xianbin Ding, Quanhua Zhou, Mei Han, Fang Wang, Lei Cheng, Hongyan Xiong

**Affiliations:** 1 Department of Epidemiology, Third Military Medical University, Chongqing, PR China; 2 Chongqing Center for Disease Control and Prevention, Chongqing, PR China; 3 Chongqing Blood Center, Chongqing, PR China

## Abstract

**Background:**

The human immunodeficiency virus type 1(HIV-1) epidemic in Chongqing, China, is increasing rapidly with the dominant subtype of CRF07_BC over the past 3 years. Since human leukocyte antigen (HLA) polymorphisms have shown strong association with susceptibility/resistance to HIV-1 infection from individuals with different ethnic backgrounds, a recent investigation on frequencies of HLA class I and class II alleles in a Chinese cohort also indicated that similar correlation existed in HIV infected individuals from several provinces in China, however, such information is unavailable in Chongqing, southwest China.

**Methods:**

In this population-based study, we performed polymerase chain reaction analysis with sequence-specific oligonucleotide probes (PCR-SSOP) for intermediate-low-resolution HLA typing in a cohort of 549 HIV-1 infected individuals, another 2475 healthy subjects from the Han nationality in Chongqing, China, were selected as population control. We compared frequencies of HLA-A, B, DRB1 alleles, haplotypes and genotypes between the two groups, and analyzed their association with HIV-1 susceptibility or resistance.

**Results:**

The genetic profile of HLA (A, B, DRB1) alleles of HIV-1 infected individuals from Chongqing Han of China was obtained. Several alleles of HLA-B such as B*46 (P = 0.001, OR = 1.38, 95%CI = 1.13-1.68), B*1501G(B62) (P = 0.013, OR = 1.42, 95%CI = 1.08-1.88), B*67 (P = 0.022, OR = 2.76, 95%CI = 1.16-6.57), B*37 (P = 0.014, OR = 1.93, 95%CI = 1.14-3.28) and B*52 (P = 0.038, OR = 1.64, 95%CI = 1.03-2.61) were observed to have association with susceptibility to HIV-1 infection in this population. In addition, the haplotype analysis revealed that A*11-B*46, A*24-B*54 and A*01-B*37 for 2-locus, and A*11-B*46-DRB1*09, A*02-B*46-DRB1*08, A*11-B*4001G-DRB1*15, A*02-B*4001G-DRB1*04, A*11-B*46-DRB1*08 and A*02-B*4001G-DRB1*12 for 3-locus had significantly overrepresented in HIV-1 infected individuals, whereas A*11-B*1502G, A*11-B*1502G-DRB1*12 and A*33-B*58-DRB1*13 were underrepresented. However, the low-resolution homozygosity of HLA-A, B, DRB1 loci and HLA-Bw4/Bw6 genotypes did not differ significantly between the two groups.

**Conclusion:**

These results may contribute to the database of HLA profiles in HIV-1 infected Chinese population, consequently, the association of certain HLA alleles with susceptibility or resistance to HIV-1 infection would provide with clues in choosing proper preventive strategies against HIV-1 infection and developing effective HIV-1 vaccines in Chinese population, especially for those in southwest China.

## Background

The HIV/AIDS epidemic remains a significant global problem because drug resistances occur so frequently and no effective preventive vaccine is available. Currently there are an estimated 33 million people living with HIV/AIDS worldwide and this number is growing rapidly, especially in developing countries where approximately 90% of the total patients lives [1]. In Asia, an estimated 4.9 million [3.7 million-6.7 million] people were living with HIV in 2007, including 440 000 [210 000-1.0 million] people who became newly infected in that year. Approximately 300 000 [250 000-470 000] patients died from AIDS-related illnesses in 2007 [2]. Chongqing, a municipal city in southwest China, has a population of more than 31 million. The first HIV-1 infected case from Chongqing was reported in 1993, by the end of 2007, up to 3913 individuals were reported as HIV-1 positive, the dominant HIV-1 subtype in Chongqing is CRF07_BC [3,4].

The human major histocompatibility complex (MHC) gene cluster on the short arm of chromosome 6, contains the most polymorphic loci in humans, the human leukocyte antigens (HLA) class I and class II genes. The class I loci, HLA-A, -B, and -C, are involved in cytotoxic T cell-mediated immunity; the class II loci, HLA-DR, -DQ, and -DP, play a role in T-helper cell-mediated immunity [5]. It has been reported that a variety of HLA alleles frequencies have been identified among various ethnic population, while several HIV-1 clades are circulating at different geographic regions. It has been shown HLA polymorphisms are associated with the susceptibility/resistance of HIV-1 infection, and have influence on the rate of disease progression of HIV/AIDS in individuals of different ethnic backgrounds [5-8]. Generally, certain HLA alleles such as B*27 and B*57, are associated with better disease outcomes, whereas other HLA alleles such as B*35 and Cw*04, can negatively affect its outcomes [5]. The association of HLA with HIV/AIDS has been studied extensively within Caucasoids and Africans, who exposed to HIV-1 subtype B or C. However, limited information is available in Asia, where a large population are at risk of HIV-1 exposures [2,8-13]. HLA-B40 and HLA-DR2 have been reported to correlate with susceptibility to HIV-1 infection in South India [9]. In China, a previous investigation of HLA-A, B, Cw alleles in HIV-1/AIDS patients from the Yi ethnic group in Sichuan province, China, showed that HLA-B*46 may be associated with susceptibility to HIV-1 infection [11], whereas HLA-A*3601, Cw*14 and Cw*0304 may correlate with slow AIDS disease progress [10]; in contrast to the B*15 allele that has been reported to accelerate disease progression in North China [12]. More recently, in another study within 43 HIV-1 seropositive Chinese patients, HLA-Bw6 homozygosity is recognized to be associated with accelerated disease progression, while HLA-Bw4 homozygosity may have a protective role [13]. Therefore, investigation on frequencies of HLA alleles and haplotypes in HIV infected individuals of different ethnic backgrounds may benefit in understanding how host genetic factors are involved in susceptibility to HIV infection, and may subsequently help in designing preventive strategies in these populations.

Hence, we hypothesized that the distribution of certain HLA alleles, haplotypes and genotypes in HIV-1 positive individuals from Chongqing, China, are different from that in the healthy population. For this purpose, we investigated frequencies of HLA-A, B, DRB1 alleles in a cohort of 549 HIV-1 infected individuals of the Han nationality from Chongqing, and compared with those frequencies from 2475 HIV negative individuals as population control. These data would contribute to the database of HLA alleles, haplotypes and genotypes distribution and their associations with HIV-1 infection in Chinese population.

## Methods

### Study Population

A total of 549 HIV-1 positive individuals, over 60% infected with CRF07_BC subtype, were enrolled in this study. Their serostatuses were tested with commercial EIA kits (BioMerieux, the Netherlands), confirmed by Western blot assay (HIV BLOT 2.2, MP Diagnostics, Singapore Science Park, Singapore) from 2002 to 2007 at Chongqing Center for Disease Control and Prevention, Chongqing, China. The participants aged from 20 to 64 years (male: female = 436:113). Among these subjects, 40.4% (222/549) was infected through heterosexual transmission, 37.0% (203/549) was infected via injecting drug use, 13.8% (76/549) through homosexual transmission, 2.7% (15/549) was via illegally commercial blood and plasma collection, along with 6.0% (33/549) via unknown routes. The population control consisted of 2475 unrelated healthy donors(18-55 years old, male: female = 1034:1441), all native Han people that have lived in Chongqing for more than three generations, selected from Chongqing Blood Center, and excluded HIV infections by assays for HIV-1 and HIV-2 antibodies. To minimize genetic variations in two groups, all participants were from the same geographic regions and ethnic group. In comparison with the population control group, more male and less female patients were recruited in the HIV-1 positive group, however, no significant difference in the mean age was identified between the two groups (38.09 ± 9.89 vs. 37.43 ± 8.58 years). Specimens were collected with informed consent under the institutionally approved protocol. Ethical approval (IRB00002276) was provided by National Center for AIDS Prevention and Control (NCAIDS) Ethical Review Board, China CDC.

### HLA Typing

DNA samples were extracted from whole blood using the TIANamp Blood DNA Kit (TIANGEN BIOTECH, Beijing, China), stored at -20°C in hydration solution before HLA typing. HLA class I and class II genotypes were determined with LABType^®^SSO Typing Test Kit (Lambda Array Beads Multi-Analyte System, One Lambda, Inc., Canoga Park, CA), using intermediate-low- resolution PCR-SSOP HLA (A, B, DRB1) genotyping, according to the manufacturer's instructions. Alleles were expressed as their 2-digit or 4-digit specificities, subsequently translated into specificity according to The HLA dictionary 2008 [14].

### Statistical Analysis

In this study, allele and haplotype frequencies were analyzed and estimated by means of the expectation maximization (EM) algorithm, exact tests of the Hardy-Weinberg equilibrium were performed using Arlequin software (ver.2.000) [15]. The configuration and frequencies of multilocus haplotypes were estimated under an unknown gametic phase.

The frequencies of HLA alleles and haplotypes between the HIV-1 positive and population control groups were compared by using a Chi-square test with Yates' correction by Epi Info 2002. Then the potential correlation of these alleles with susceptibility or resistance to HIV-1 infection was evaluated by a binary logistic regression analysis with multivariate comparisons by SPSS14.0. The strength of an association with haplotypes and genotypes, calculated with Epi Info, was indicated by an odds ratio (OR) with a 95% confidence interval (CI) and P value, which was corrected by the false discovery rate (FDR) method for multiple testing. The FDR correction is defined in step-up fashion with the equation of "s_R _= p_R_, s_(R-1) _= min (s_R_, [R/(R-1)]p_(R-1)_), s_(R-2) _= min (s_(R-2)_, [R/(R-2)]p_(R-2)_), ..." to control the false discovery rate by "false discovery rate" described by Benjamini and Hochberg. P value less than 0.05 level was considered significant in multivariate analysis.

## Results

### Frequencies of HLA (A, B, DRB1) allele in HIV-1 positive and population control groups

First we detected frequencies of each HLA allele in the two groups. Frequencies of all identified HLA (A, B, DRB1) alleles in 549 HIV-1 infected individuals and those in the control group are listed in Table 1. In the HIV-1 positive group, the predominant alleles of the 13 HLA-A types detected with a frequency of >10%, were A*11, A*02 and A*24, consisting of more than 80% of the total alleles. Among the 29 HLA-B alleles identified, the most common types in a descending order were: B*46, B*4001G (B60), B*13 and B*1501G (B62), etc. At the DRB1 locus, DRB1* 09, DRB1* 15, DRB1* 12, DRB1* 04 and DRB1* 14 were determined with a frequency of greater than 10% among the 13 HLA-DRB1 alleles detected totally. Meanwhile, in the population control group, dominant alleles at the HLA-A locus were A*11, A*02 and A*24, exactly the same alleles as those in the infected group. The top 4 HLA-B alleles were B*46, B*4001G (B60), B*13 and B*58, while alleles at the DRB1 locus with higher frequencies were as follows: DRB1* 09, DRB1* 15, DRB1*12 and DRB1*04. Preliminarily, only the frequency of B*46 allele was observed with significant difference between the two groups (P < 0.05). In addition, frequencies observed in all alleles did not deviate significantly from Hardy-Weinberg equilibrium, P values for A(0.99 vs 0.30), B(0.26 vs 0.51), DRB1(0.45 vs 0.64) allele frequencies in both groups were greater than 0.05.

**Table 1:** Frequencies of HLA (A, B, DRB1) alleles in HIV-1 positive and population control groups

		Allele frequency				Allele frequency	
					
Allele	Specificity	HIV-1 Positive	Population Control	Allele	specificity	HIV-1 Positive	Population Control
B*13	B13	0.0929	0.0994			0.0246	0.0184
B*1501G	B62	0.0701	0.0568	A*11	A11	0.3215	0.3259
B*1502G	B75	0.0537	0.0659	A*02	A2	0.3133	0.3014
B*1509G	B70	0.0046	0.0055	A*24	A24	0.1740	0.1810
B*1517G	B63	0.0018	0.0008	A*26	A26	0.0264	0.0263
B*1512G	B76	0.0046	0.0030	A*29	A29	0.0064	0.0040
B*15xx	B15	0.0009	0.0002	A*03	A3	0.0091	0.0139
B*18	B18	0.0018	0.0040	A*30	A30	0.0200	0.0222
B*27	B27	0.0100	0.0115	A*31	A31	0.0228	0.0196
B*35	B35	0.0319	0.0368	A*32	A32	0.0046	0.0051
B*37	B37	0.0182	0.0109	A*33	A33	0.0710	0.0762
B*38	B38	0.0328	0.0331	A*68	A68	0.0055	0.0032
B*39	B39	0.0191	0.0226	A*74	A74	0.0009	0.0006
B*4001G	B60	0.1348	0.1483	Others			0.0022
B*4002G	B61	0.0383	0.0362				
B*44	B44	0.0191	0.0184				
B*46^▲^	**B46**	**0.2077**	**0.1741**	DRB1* 01	DR1	0.0155	0.0160
B*48	B48	0.0100	0.0123	DRB1*10	DR10	0.0200	0.0164
B*50	B50	0.0009	0.0012	DRB1* 11	DR11	0.0647	0.0614
B*51	B51	0.0528	0.0612	DRB1* 12	DR12	0.1075	0.1271
B*52	B52	0.0228	0.0158	DRB1* 13	DR13	0.0483	0.0588
B*54	B54	0.0364	0.0291	DRB1* 14	DR14	0.1011	0.0820
B*55	B55	0.0364	0.0382	DRB1* 15	DR15	0.1266	0.1297
B*56	B56	0.0109	0.0077	DRB1* 16	DR16	0.0446	0.0432
B*57	B57	0.0046	0.0077	DRB1* 03	DR17	0.0492	0.0469
B*58	B58	0.0610	0.0709	DRB1* 04	DR4	0.1020	0.1030
B*67	B67	0.0073	0.0030	DRB1* 07	DR7	0.0383	0.0436
B*07	B7	0.0118	0.0160	DRB1* 08	DR8	0.0847	0.0792
B*08	B8	0.0027	0.0044	DRB1* 09	DR9	0.1976	0.1921
Others			0.0050	Others			0.0006

^▲^: indicates P value < 0.05 of comparative frequencies in the HIV-1 positive and population control group. P value has been adjusted with Yates' correction.

### Frequencies of 2-locus HLA-A-B haplotypes and 3-locus HLA-A-B-DRB1 haplotypes in HIV-1 positive and population control groups

After frequencies of the HLA alleles in the two groups were determined, the distribution of specific 2-locus or 3-locus haplotypes would provide with more detailed HLA genetic profiles in both groups. Hundreds of 2-locus and 3-locus haplotypes were obtained, those haplotypes with relatively higher frequencies were shown in Table 2. For the 2-locus HLA-A-B haplotypes, the most common haplotypes in the HIV-1 positive group were A*02-B*46, A*11-B*4001G, A*33-B*58, A*11-B*46, A*11-B*13. In the population control group, however, HLA-A-B haplotypes with higher frequencies were A*02-B*46, A*11-B*4001G, A*33-B*58, A*11-B*1502G, A*24-B*4001G, respectively. Meanwhile, for the 3-locus haplotypes of HLA-A-B-DRB1, haplotypes of A*02-B*46-DRB1*09, A*11-B*46-DRB1*09 and A*33-B*58-DRB1*03 in the HIV-1 positive group, and haplotypes of A*02-B*46-DRB1*09, A*33-B*58-DRB1*03 and A*11-B*1502G-DRB1*12 in the population control group, were more frequently observed. Consequently, significant differences of frequencies were identified between the two groups in four 2-locus haplotypes and eight 3-locus haplotypes (Table 2).

**Table 2:** Distribution of HLA haplotypes in HIV-1 positive and population control groups

HIV-1 positive		Population control		HIV-1 positive		Population control	
			
(2-locus) Haplotype	Frequency	(2-locus) Haplotype	Frequency	(3-locus) Haplotype	Frequency	(3-locus) Haplotype	Frequency
A*02 B*46	0.1437	A*02 B*46	0.1230	A*02 B*46 DRB1*09	0.0640	A*02 B*46 DRB1*09	0.0659
A*11 B*4001G	0.0597	A*11 B*4001G	0.0651	A*11 B*46 DRB1*09^▲^	0.0316	A*33 B*58 DRB1*03	0.0306
A*33 B*58	0.0470	A*33 B*58	0.0569	A*33 B*58 DRB1*03	0.0282	A*11 B*1502G DRB*12^▲^	0.0255
A*11 B*46^▲^	0.0463	A*11 B*1502G^▲^	0.0454	A*02 B*46 DRB1*08^▲^	0.0274	A*11 B*13 DRB1*15	0.0218
A*11 B*13	0.0448	A*24 B*4001G	0.0447	A*02 B*46 DRB1*14	0.0230	A*11 B*46 DRB1*09	0.0186
A*02 B*4001G	0.0414	A*11 B*13	0.0437	A*11 B*13 DRB1*15	0.0208	A*33 B*58 DRB1*13^▲^	0.0174
A*24 B*4001G	0.0300	A*02 B*4001G	0.0312	A*11 B*4001G DRB1*15^▲^	0.0194	A*02 B*46 DRB1*08	0.0167
A*11 B*1501G	0.0293	A*11 B*46	0.0296	A*30 B*13 DRB1*07	0.0161	A*02 B*46 DRB1*14	0.0152
A*11 B*1502G	0.0281	A*11 B*1501G	0.0231	A*11 B*1501G DRB1*04	0.0160	A*30 B*13 DRB1*07	0.0147
A*24 B*54^▲^	0.0234	A*11 B*51	0.0222	A*01 B*37 DRB1*10	0.0118	A*11 B*4001G DRB1*12	0.0137
A*11 B*51	0.0224	A*02 B*38	0.0218	A*02 B*4001G DRB1*09	0.0112	A*11 B*4001G DRB1*04	0.0120
A*30 B*13	0.0186	A*30 B*13	0.0191	A*02 B*4001G DRB1*04^▲^	0.0103	A*11 B*1501G DRB1*04	0.0104
A*02 B*13	0.0171	A*02 B*13	0.0189	A*24 B*54 DRB1*04	0.0102	A*11 B*4001G DRB1*09	0.0097
A*24 B*1501G	0.0163	A*11 B*55	0.0167	A*11 B*46 DRB1*08^▲^	0.0096	A*11 B*1502G DRB1*15	0.0093
A*02 B*55	0.0160	A*24 B*13	0.0165	A*11 B*4001G DRB1*09	0.0096	A*11 B*13 DRB1*12	0.0088
A*02 B*1501G	0.0153	A*24 B*1501G	0.0151	A*11 B*4001G DRB1*12	0.0089	A*11 B*4001G DRB1*08	0.0085
A*02 B*38	0.0151	A*24 B*46	0.0145	A*02 B*46 DRB1*04	0.0087	A*24 B*4001G DRB1*09	0.0085
A*24 B*46	0.0146	A*24 B*51	0.0137	A*02 B*4001G DRB1*12^▲^	0.0085	A*24 B*4001G DRB1*15	0.0079
A*24 B*4002G	0.0145	A*02 B*51	0.0132	A*24 B*46 DRB1*09	0.0084	A*11 B*51 DRB1*09	0.0079
A*01 B*37^▲^	0.0145	A*02 B*1502G	0.0122	A*02 B*38 DRB1*16	0.0081	A*02 B*38 DRB1*16	0.0070

Only the top 20 most common haplotypes are listed.

^▲^: indicates P value < 0.05 of comparative frequencies in the HIV-1 positive and population control group. P value has been adjusted with Yates' correction.

### Multivariate analysis

A binary logistic regression analysis of forward stepwise (wald) method with multivariate comparisons was conducted for all alleles listed in Table 1, at HLA-A, B, DRB1 locus, respectively. For HLA-A and DRB1 alleles, no significant statistical difference was identified. However, besides the B*46 allele that has demonstrated a significant difference as above mentioned, other B alleles such as B*1501G (B62), B*37, B*67, B*52 independently had showed association with HIV-1 susceptibility in the Chongqing Han(Table 3), as indicated by a significant P value and OR in the multivariate logistic regression analysis.

**Table 3:** Binary logistic regression analysis

				95.0% C.I^c ^for Exp(B)	
				
	B coefficient	P value^a^	Exp(B).^b^	lower	Upper
B*46	**0.322**	**0.001**	**1.380**	**1.133**	**1.681**
B*37	**0.660**	**0.014**	**1.934**	**1.140**	**3.281**
B*1501G	**0.352**	**0.013**	**1.422**	**1.078**	**1.877**
B*67	**1.014**	**0.022**	**2.757**	**1.156**	**6.574**
B*52	**0.493**	**0.038**	**1.638**	**1.029**	**2.607**
B*54	0.352	0.060	1.422	0.985	2.053
Constant	-1.733	0.000	0.177		

Multivariate logistic regression analysis: Covariates (all HLA-A, HLA-B, HLA-DRB1alleles in Table 1 entered independently); Method = Forward Wald; Probability for stepwise = Entry (0.1), Remove (0.2).

^a^P value, Significance; ^b^Exp(B), Exponent of B, odds ratio; ^c^CI, Confidence interval.

### Comparison of HLA haplotypes and genotype distribution in HIV-1 positive and population control groups

Next, we analyzed and compared those 2- and 3-locus haplotypes with significantly different frequencies (P < 0.05) between the HIV-1 positive group and population control group. The statistical difference in the presence of a haplotype or a genotype between the two groups was assessed with an OR and 95%CI, P values were reported as both uncorrected and corrected for multiple comparisons (Table 4). For 2-locus haplotypes, four of them were significantly different in the HIV-1 positive group: A*11-B*46, A*24-B*54 and A*01-B*37 were overrepresented in the HIV-1 positive group. Meanwhile, another haplotype, A*11-B*1502G was significantly lower. For 3-locus haplotypes of HLA-A-B-DRB1, 6 out of the top 20 haplotypes were overrepresented in the HIV-1 positive group: A*11-B*46-DRB1*09, A*02-B*46-DRB1*08, A*11-B*4001G- DRB1*15, A*02-B*4001G-DRB1*04, A*11-B*46-DRB1*08 and A*02-B*4001G-DRB1*12. On the contrary, another 2 haplotypes, A*11-B*1502G-DRB1*12 and A*33-B*58-DRB1*13 haplotypes were underrepresented in the HIV-1 positive group.

**Table 4:** Comparison of HLA haplotypes and genotypes in HIV-1 positive and population control groups

Haplotype/Genotype	No. of HIV-1 Positive N (%)	No. of Population Control N (%)	P value	Corrected P value	Odds ratios	95%CI
2-locus Haplotype						
A*11 B*46^▲^	**50 (9.1)**	**143 (5.8)**	**0.005**	**0.012**	**1.630**	**1.15-2.32**
A*24 B*54^▲^	**26 (4.7)**	**57 (2.3)**	**0.003**	**0.012**	**2.110**	**1.28-3.46**
A*01 B*37^▲^	**16 (2.9)**	**34 (1.4)**	**0.017**	**0.017**	**2.160**	**1.13-4.07**
A*11 B*1502G^▲^	**30 (5.5)**	**220 (8.9)**	**0.011**	**0.012**	**0.590**	**0.39-0.89**
3-locus Haplotype						
A*11 B*46 DRB1*09^▲^	**35 (6.4)**	**92 (3.7)**	**0.007**	**0.032**	**1.760**	**1.16-2.68**
A*02 B*46 DRB1*08^▲^	**30 (5.5)**	**82 (3.3)**	**0.022**	**0.038**	**1.690**	**1.07-2.64**
A*11 B*4001G DRB1*15^▲^	**21 (3.8)**	**29 (1.2)**	**<0.0001**	**0.0007**	**3.350**	**1.83-6.13**
A*02 B*4001G DRB1*04^▲^	**11 (2.0)**	**17 (0.7)**	**0.008**	**0.032**	**2.960**	**1.29-6.70**
A*11 B*46 DRB1*08^▲^	**11 (2.0)**	**9 (0.4)**	**<0.0001**	**0.0007**	**5.600**	**2.15-14.74**
A*02 B*4001G DRB1*12^▲^	**9 (1.6)**	**16 (0.6)**	**0.039**	**0.039**	**2.560**	**1.04-6.17**
A*11 B*1502G DRB1*12^▲^	**7 (1.3)**	**126 (5.1)**	**0.0001**	**0.0007**	**0.240**	**0.10-0.54**
A*33 B*58 DRB1*13^▲^	**9 (1.6)**	**86 (3.5)**	**0.036**	**0.039**	**0.460**	**0.22-0.96**
Homozygosity						
A-A	120 (21.9)	590 (23.8)	0.96	0.96	1.00	0.73-1.35
B-B	43 (7.8)	198 (8.0)	0.23	0.35	0.89	0.73-1.08
DRB1-DRB1	61 (11.1)	265 (10.7)	0.23	0.35	1.13	0.93-1.36
Serological group						
Bw4-Bw4	61 (11.1)	276 (11.1)	0.35	0.96	0.89	0.71-1.12
Bw6-Bw6	226 (41.2)	1091 (44.1)	0.96	0.96	0.98	0.68-1.40
Bw4-Bw6	262 (47.7)	1108 (44.8)	0.84	0.96	1.04	0.77-1.41

"N" indicates the number of people with a given haplotype/genotype. The percentage is indicated in parentheses; P value with Yates' correction; CI, confidence interval.

^▲^: indicates P value and corrected P value (adjusted with False discovery rate for multiple testing) <0.05 of comparative distribution of haplotypes and genotypes in the HIV-1 positive and population control group.

In addition, considering the possible role of homozygosity and Bw4/Bw6 serotype on HIV-1 infection, we also compared the HLA-A, B, DRB1 homozygotes and HLA-Bw4, Bw6 serological types in these two groups. However, the homozygosity of A-A, B-B, DRB1-DRB1 and the serotype group of Bw4-Bw4, Bw4-Bw6, Bw6-Bw6 from this population revealed no significant difference between the two groups.

## Discussion

In this study, 13 alleles of HLA-A, 29 of HLA-B, 13 of HLA-DRB1, respectively, were identified among HIV-1 infected individuals of the Han nationality from Chongqing, China. Generally, the distribution of HLA-A, B, DRB1 alleles in HIV-1 infected individuals and population control was similar to other populations in China, much closer to Southern Han than to Northern Han of China [16,17]. To investigate associations between HLA alleles and susceptibility/resistance to HIV infection, we compared the distribution of each allele between the two groups. Although the frequencies of A*01, B*54, and DRB1*14 were overrepresented, the frequencies of A*03, B*58, B*1502G (B75), DRB1*12, DRB1*13 were lower in HIV-1 positive group, overall, there was no statistically significant difference. Meanwhile, B*46, B*1501G (B62), B*37, B*67 and B*52 alleles were observed to be disadvantageous factor from HIV-1 infection, suggesting that these five alleles may be considered as risk factors that increased the susceptibility of HIV-1 infection in Chongqing Han.

The allele of HLA-B*46, which is rare or infrequent in white and black population, but with a higher frequency in Southern Han population than that in Northern Han, is the most common HLA-B allele in Chongqing Han [16-18]. This allele has been reported to be associated with the susceptibility to HIV-1 infections in the Yi ethnic group in Sichuan province, China [11]. Our results were in consistent with that observation, suggesting that B*46 may be associated with increased susceptibility to HIV-1 infections in Chongqing Han population as well.

The HLA-B*15 allele, which included 6 different specificities (B62, B75, B70, B63, B76, B15) in HIV-1 infected individuals, was another commonly observed allele in Chongqing Han. It has been reported that B*1501G (B62) and B*1502G (B75) were more common than others in southwest China [19]. Moreover, the B*1501G (B62) alleles, another risky alleles by the Meta-analysis with confirmed association with HIV infection and AIDS progression in certain population [20], appeared to offer risk from HIV-1 infection in the Chongqing Han population. Thus, it's not surprising that frequencies of A*11-B*1501G and A*11-B*1501G-DRB1*04 haplotypes, all containing B*1501G (B62), were higher in HIV infection group, although without significance. In contrast, the frequency of B*1502G (B75) is lower (5.37% vs. 6.59%, p = 0.15) and A*11-B*1502G, A*11-B*1502G-DRB1*12 haplotypes were statistically significantly underrepresented in the HIV-1 positive group. Taken together, our data suggested that B*1501G (B62) may be a risk factor for HIV-1 infection, whereas B*1502G (B75) may be a protective factor against HIV-1 infection. However, for all alleles of B*15(13.57% vs. 13.21%), there was no difference between the HIV-1 positive and population control group. Therefore, extensive studies with high-resolution HLA subtypes or in combination with other alleles would provide with more clues for the association of the HLA-B*15 allele polymorphisms with HIV infection [21].

The HLA-B58 supertype of structurally and functionally related alleles (B*57, B*58, B*1516 and B*1517) had been reported to be consistently associated with low HIV-1 viraemia and prolonged AIDS survival, especially in Caucasoid and African populations, whose prevalent subtype is HIV-1 B or C [5,8,22,23]. In our study, we also observed that the frequencies of B*57 (0.46% vs. 0.77%, p = 0.27), B*58 (6.10% vs. 7.09%, p = 0.27) and B58s (6.73% vs. 7.94%, p = 0.19) were lower in the HIV-1 infected individuals. Furthermore, the haplotypes of A*33-B*58 (4.70% vs. 5.69%, p = 0.23) and A*33-B*58-DRB1*13 (0.80% vs. 1.74%, p = 0.038) were underrepresented in the HIV positive group. This trend indicated the B*57 and B*58 alleles might be protective factors to HIV infection in Chongqing Han.

In this study, we noticed that B*37 allele, A*01-B*37 and A*01-B*37-DRB1*10 haplotypes showed disadvantages in HIV-1 resistance, that B*54 allele (P = 0.060, OR = 1.42, 95%CI = 0.99-2.05) correlated with risks to HIV-1 infection and A*24-B*54 haplotype showed significantly overrepresented in the HIV-1 positive individuals, and that both B*67 and B*52 alleles also showed disadvantages in HIV-1 resistance. All of these results suggested that B*67, B*37, B*52 and B*54 alleles were associated with HIV-1 susceptibility. To our knowledge, the association of B*67, B*37, B*52 and B*54 alleles with HIV-1 infection has not been reported elsewhere yet. Meanwhile, these four alleles were uncommon in Chongqing Han population. Therefore, the role of these alleles as risk factors for HIV-1 infection remains to be confirmed by more samples and extensive studies in other populations.

The HLA-B*40 alleles, which is slightly less common than the B*46 allele in Chongqing Han population, was separated into two serogroups, B*4001G (B60) and B*4002G (B61). Although neither of them showed any difference between the HIV-1 positive and population control group, several 3-locus haplotypes containing B*4001G (B60) such as A*11-B*4001G-DRB1*15, A*02-B*4001G-DRB1*04 and A*02-B*4001G-DRB1*12, were significantly overrepresented in the HIV positive group than that in the population control group, suggesting this allele might be another risk factor of HIV-1 infection in Chongqing Han, in consistent with the observation that HLA-B*40 correlated with susceptibility to HIV-1 infection in South India [9].

Among the 2-locus haplotypes, it is not surprising that the A*02-B*46 (27.0% vs. 23.7%, p = 0.11) and A*11-B*46 (9.1% vs. 5.8%, p = 0.005) were more frequent and overrepresented in the HIV-1 infected individuals. In the 3-locus haplotypes, frequencies of several common haplotypes such as A*11-B*46-DRB1*09, A*02-B*46-DRB1*08, A*02-B*46-DRB1*14, A*11-B*4001G- DRB1*15, A*02-B*4001G-DRB1*04, A*11-B*1501G-DRB1*04, A*11-B*46-DRB1*08 and A*02-B*4001G-DRB1*12 were observed to be higher in the HIV-1 positive group, suggesting these haplotypes could be susceptible factors for HIV-1 infection in Chongqing Han. Meanwhile, most haplotypes containing common alleles such as A*02, A*11, B*46, B*4001G, B*1501G, DRB1*09, DRB1*08, DRB1*15 were more frequently observed in Chongqing Han, also suggested that possession of common HLA specificity may be more susceptible to HIV-1 infection [24-26]. For the observed HLA polymorphisms on HIV transmission, it has been proposed that intense immune pressure can give rise to HIV variants that are not easily eliminated by the HLA-concordant recipient with a similar antiviral repertoire, suggesting that HLA allele match between the donor and recipient might lead to immune responses that increase the possibility of viral transmission [27]. Consequently, HIV-1 virus evolved to escape an efficient immune response mediated by common alleles in the population, but remains susceptible to responses mediated by low-frequency alleles [5,24]. These findings also demonstrated the importance of frequency-dependent effects of host genes on HIV-1 evolution in different populations, suggesting that HLA-driven viral evolution critically influences control of viraemia in early HIV-1 infection [28].

Our results also supported previous conclusions that HLA-B alleles could influence HIV infection in that most HLA-specific CTL responses were HLA-B-restricted. Moreover, immune selection pressure on HIV-1 virus was disproportionately driven by HLA-B alleles, in consistent with the observation that B alleles evolve more rapidly than other HLA alleles [8,29].

For proper interpretation of these data, remarkably, two statistical issues regarding the composition of enrolled subjects in this study should be considered. One is the sex ratio in the two groups, a preliminary study in our team revealed no significant difference between frequencies of HLA alleles and haplotypes from both male and female (data not shown), therefore, the fact that more male patients and less female patients were recruited in the HIV-1 positive group of this study was neglected in analyses mentioned above. The other one is the possibility of lead time bias that is caused by different duration of seroconversion in the participants. In this study, despite the duration of most of the HIV-1 positive individuals enrolled in this study was unknown, in fact, HIV infection was rare in Chongqing a decade ago, over 80% of current HIV-1 infected individuals was detected positive in the recent 3 years, thus, the seroconversion time is not an important issue in these subjects from Chongqing. However, to avoid such bias, duration of seroconversion in HIV-1 infected individuals should be considered in prolonged larger-scale investigations in the future. In addition, since we used a normal population, whose status of high risk behaviors for HIV infection was unavailable, as the control in this study, we could not analyze the association of HLA with HIV infection separately according to variation in transmission route. To address further association of HLA antigens with HIV-1 infection in Chongqing in the following years, if the transmission routes are considered, another cohort study, which includes normal population control with similar high risk behaviors to HIV-1 positive individuals, will be conducted with categorized risk behaviors.

## Conclusion

In summary, a distinct profile of HLA class I (A, B) and II (DRB1) alleles of HIV-1 infected individuals from Chongqing Han of China was observed. HLA polymorphism of the HIV-1 positive group, such as the alleles of B*46, B*1501G (B62), B*67, B*37, B*52 and haplotypes of A*24-B*54, A*11-B*1502G, A*11-B*1502G-DRB1*12, A*33-B*58-DRB1*13 and certain common haplotypes provided constructive information for further study to determine their associations with HIV-1 infection. These data contributed to database of HLA polymorphisms of HIV-1 infected individuals in the Chinese population, provided with genetic clues for designing effective vaccines, and for choosing proper prevention and treatment strategies of HIV/AIDS for Han ethnic of China, or specifically in southwest China.

## Competing interests

The authors declare that they have no competing interests.

## Authors' contributions

HX designed this study and helped with interpretation of results and preparation of the manuscript. XH drafted the manuscript, carried out the HLA genotyping assays and analyzed the data. HL contributed to acquisition of sample/data and the manuscript revision. XD, QZ and MH recruited the HIV-1 infected individuals for the study. WM, FW, and LC contributed to acquisition of the data of population control. All authors read and approved the final manuscript.

## Pre-publication history

The pre-publication history for this paper can be accessed here:

http://www.biomedcentral.com/1471-2334/9/201/prepub
